# Women’s, partners’ and healthcare providers’ views and experiences of assisted vaginal birth: a systematic mixed methods review

**DOI:** 10.1186/s12978-020-00915-w

**Published:** 2020-06-01

**Authors:** Nicola Crossland, Carol Kingdon, Marie-Clare Balaam, Ana Pilar Betrán, Soo Downe

**Affiliations:** 1 Faculty of Health and Wellbeing, University of Central Lancashire, Preston, PR1 2HE UK; 2 Research in Childbirth and Health Unit, University of Central Lancashire, Preston, PR1 2HE UK; 3 UNDP/UNFPA/UNICEF/WHO/World Bank Special Programme of Research, Development and Research Training in Human Reproduction (HRP), Department of Reproductive Health and Research, World Health Organization, Geneva, Switzerland

**Keywords:** Assisted vaginal delivery, Instrumental delivery, Operative delivery, Ventouse, Vacuum extraction, Forceps delivery, Childbirth, Caesarean section, Evidence synthesis

## Abstract

**Background:**

When certain complications arise during the second stage of labour, assisted vaginal delivery (AVD), a vaginal birth with forceps or vacuum extractor, can effectively improve outcomes by ending prolonged labour or by ensuring rapid birth in response to maternal or fetal compromise. In recent decades, the use of AVD has decreased in many settings in favour of caesarean section (CS). This review aimed to improve understanding of experiences, barriers and facilitators for AVD use.

**Methods:**

Systematic searches of eight databases using predefined search terms to identify studies reporting views and experiences of maternity service users, their partners, health care providers, policymakers, and funders in relation to AVD. Relevant studies were assessed for methodological quality. Qualitative findings were synthesised using a meta-ethnographic approach. Confidence in review findings was assessed using GRADE CERQual. Findings from quantitative studies were synthesised narratively and assessed using an adaptation of CERQual. Qualitative and quantitative review findings were triangulated using a convergence coding matrix.

**Results:**

Forty-two studies (published 1985–2019) were included: six qualitative, one mixed-method and 35 quantitative. Thirty-five were from high-income countries, and seven from LMIC settings. Confidence in the findings was moderate or low. Spontaneous vaginal birth was most likely to be associated with positive short and long-term outcomes, and emergency CS least likely. Views and experiences of AVD tended to fall somewhere between these two extremes. Where indicated, AVD can be an effective, acceptable alternative to caesarean section. There was agreement or partial agreement across qualitative studies and surveys that the experience of AVD is impacted by the unexpected nature of events and, particularly in high-income settings, unmet expectations. Positive relationships, good communication, involvement in decision-making, and (believing in) the reason for intervention were important mediators of birth experience. Professional attitudes and skills (development) were simultaneously barriers and facilitators of AVD in quantitative studies.

**Conclusions:**

Information, positive interaction and communication with providers and respectful care are facilitators for acceptance of AVD. Barriers include lack of training and skills for decision-making and use of instruments.

## Abstrait

**Contexte:**

Lors de complications au cours du deuxième stade du travail, l’utilisation de forceps ou d’une ventouse peut améliorer l’issue de l’accouchement par voie basse en assurant une naissance rapide lorsque la mère ou le fœtus se trouvent en difficulté. Au cours des dernières décennies, l’utilisation de l’accouchement assisté par voie basse a diminué dans de nombreuses régions en faveur de la césarienne. Cette revue vise à mieux comprendre les expériences et les facteurs qui facilitent ou empêchent l’utilisation de l’accouchement assisté par voie basse.

**Méthodes:**

Recherches systématiques dans huit bases de données à l’aide de termes de recherche prédéfinis pour identifier les études rapportant les points de vue et les expériences des utilisatrices de services de maternité, de leurs partenaires, des prestataires de soins de santé, des responsables politiques et des bailleurs de fonds en rapport avec l’accouchement assisté par voie basse. La qualité méthodologique des études pertinentes a été évaluée. Les résultats qualitatifs ont été synthétisés à l’aide d’une approche méta-ethnographique. La confiance envers les résultats de l’examen a été évaluée à l’aide de l’approche GRADE CERQual. Les résultats des études quantitatives ont été synthétisés de manière narrative et évalués à l’aide d’une adaptation de CERQual. Les résultats des examens qualitatifs et quantitatifs ont été triangulés à l’aide d’une matrice de codage des convergences.

**Résultats:**

42 études (publiées de 1985 à 2019) ont été incluses: six qualitatives, une mixte et 35 quantitatives. Trente-cinq provenaient de pays à revenus élevés et sept de pays à revenus faibles ou intermédiaires. La confiance envers les résultats était modérée ou faible. L’accouchement spontané par voie basse était le plus susceptible d’être associé à des résultats positifs à court et à long terme, et la césarienne d’urgence la moins susceptible de l’être. Les opinions et les expériences relatives à l’accouchement assisté par voie basse se situaient généralement entre ces deux extrêmes. Sur indication médicale, l’accouchement assisté par voie basse peut être une alternative efficace et acceptable à la césarienne. Les études qualitatives et les enquêtes s’accordent de façon totale ou partielle sur le fait que l’expérience de l’accouchement assisté par voie basse est. affectée par la nature inattendue des événements et, en particulier dans les pays à revenu élevé, les attentes non satisfaites. Des relations positives, une bonne communication, une participation à la prise de décision et (une foi en) la raison de l’intervention étaient d’importants médiateurs de l’expérience de l’accouchement. Les attitudes et (le développement des) compétences professionnelles étaient simultanément des obstacles et des facilitateurs de l’accouchement assisté par voie basse dans les études quantitatives.

**Conclusion:**

L’information, l’interaction positive et la communication avec les prestataires ainsi que les soins respectueux facilitent l’acceptation de l’accouchement assisté par voie basse. Les obstacles comprennent le manque de formation et de compétences pour la prise de décision et l’utilisation d’instruments.

## Resumen

**Antecedentes:**

Cuando surgen ciertas complicaciones durante la segunda etapa del parto, el parto vaginal asistido, es decir, un parto vaginal con fórceps o ventosa, puede mejorar efectivamente los resultados al poner fin a un parto prolongado o asegurar un parto más rápido en caso de riesgo para la madre o el feto. En las últimas décadas, el uso del parto vaginal asistido ha disminuido en muchos entornos en favor de la cesárea. Esta revisión tuvo como objetivo mejorar la comprensión de las experiencias, los obstáculos y los elementos facilitadores para el uso del parto vaginal asistido.

**Métodos:**

Búsquedas sistemáticas en ocho bases de datos utilizando términos de búsqueda predefinidos para identificar estudios que aportaran puntos de vista y experiencias de usuarias de servicios de maternidad, sus parejas, proveedores de atención médica, responsables de la formulación de políticas y entidades financiadoras en relación con el parto vaginal asistido. Se evaluó la calidad metodológica de los estudios. Los hallazgos cualitativos se sintetizaron utilizando un enfoque meta-etnográfico y la confianza en los resultados se evaluó mediante GRADE CERQual. Los resultados de los estudios cuantitativos se sintetizaron narrativamente y se evaluaron mediante una adaptación de CERQual. Los resultados de la revisión cualitativa y cuantitativa se triangularon utilizando una matriz de codificación de convergencia.

**Resultados:**

Se incluyeron 42 estudios (publicados entre 1985 y 2019): seis cualitativos, uno mixto y 35 cuantitativos. Treinta y cinco procedían de países de altos ingresos y siete de entornos pertenecientes a países de ingresos bajos y medios. La confianza en los resultados fue moderada o baja. El parto vaginal espontáneo era el que tendía a estar más asociado con resultados positivos a corto y largo plazo, y la cesárea de emergencia la que menos lo estaba. Las opiniones y experiencias del parto vaginal asistido se encontraban en un lugar intermedio entre los anteriores. El parto vaginal asistido, cuando está indicado, puede ser una alternativa efectiva y aceptable a la cesárea. Los estudios y encuestas de índole cualitativa convinieron, total o parcialmente, en que la experiencia del parto vaginal asistido se ve afectada por el carácter inesperado de los acontecimientos y, especialmente en entornos de altos ingresos, por las expectativas no satisfechas. Las relaciones positivas, la buena comunicación, la participación en la toma de decisiones y (creer en) el motivo de la intervención fueron mediadores importantes en la experiencia del parto. Las actitudes y habilidades profesionales fueron al mismo tiempo obstáculos y facilitadores del parto vaginal asistido en estudios cuantitativos.

**Conclusiones:**

La información, la interacción positiva y la comunicación con los proveedores, así como la atención respetuosa, son facilitadores para la aceptación del parto vaginal asistido. Los obstáculos incluyen la falta de capacitación y de habilidades para la toma de decisiones y para el uso de los instrumentos.

## Resumo

**Contexto:**

Quando surgem algumas complicações no segundo período do trabalho de parto, o parto vaginal instrumental (PVI), a fórcipe ou com vácuo extrator, pode melhorar os desfechos. Isso se dá porque o PVI pode encurtar o trabalho de parto prolongado ou acelerar o parto no caso de complicações maternas ou fetais. Nas últimas décadas, o uso do PVI tem diminuído em muitos locais devido à preferência pela cesariana (CS). O objetivo desta revisão foi ampliar o conhecimento sobre as experiências, as barreiras, e os facilitadores para o uso do PVI.

**Métodos:**

Fizemos uma busca sistematizada em oito bases de dados usando palavras pré-definidas para identificar estudos com dados sobre as opiniões e experiências de usuárias de maternidades, seus parceiros, profissionais de saúde, formuladores de políticas, e financiadores sobre o PVI. Avaliamos a qualidade metodológica dos estudos incluídos. Usamos a abordagem meta-etnográfica para fazer uma síntese dos achados qualitativos. Usamos o GRADE CERQual para avaliar a confiança nos resultados da revisão. Usamos uma adaptação do GRADE CERQual para sintetizar os resultados dos estudos quantitativos. Triangulamos os resultados qualitativos e quantitativos da revisão usando uma matriz de convergência dos modos de codificação.

**Resultados:**

Incluímos 42 estudos (publicados entre 1985–2019): seis qualitativos, um estudo com métodos mistos e 35 estudos quantitativos. Trinta e cinco estudos eram de países de alta renda e sete eram de países de baixa ou média renda. A confiança nos resultados foi moderada ou baixa. O parto vaginal espontâneo foi a via de parto com maior probabilidade de desfechos positivos no curto e no longo prazo enquanto a CS de emergência foi a via com menor probabilidade desses desfechos. As opiniões e experiências relacionadas ao PVI ficaram entre esses dois extremos. Quando indicado, o PVI pode ser uma alternativa eficaz e aceitável à cesariana. Nos estudos e inquéritos qualitativos, houve concordância total ou parcial que a experiência do PVI é afetada pela natureza inesperada dos eventos e por expectativas frustradas, especialmente nos países de alta renda. Relações positivas, uma boa comunicação, o envolvimento na tomada de decisões, e acreditar na indicação do procedimento foram importantes mediadores da experiência do parto. Nos estudos quantitativos, a atitude e a competência dos profissionais (desenvolvimento) foram tanto barreiras como facilitadores para o PVI.

**Conclusões:**

Informações, interações e comunicação positivas com os profissionais de saúde, e uma assistência respeitosa são facilitadores para a aceitação do PVI. As barreiras incluem a falta de treinamento e competência para a tomada de decisões, além do uso de instrumentos.

## Plain English summary

Assisted vaginal delivery (AVD) is a vaginal birth where an instrument, usually forceps or vacuum extractor, is used to help the birth if complications arise during the second stage of labour. In many countries, AVD has become less commonly used and rates of caesarean section (CS) have risen. While CS can be life-saving for mother or baby, it is sometimes used where there is no medical need, which has risks. It is possible that AVD could be used in some situations instead of unnecessary CS. AVD is safe when used properly but has risks if used inappropriately or by unskilled people. Our aim in this review was to explore parents’ and healthcare providers’ views and experiences of AVD to understand what might support or prevent its use. We reviewed 42 studies (published 1985–2019), 35 from high-income countries, and seven from low and middle-income countries. We rated the confidence in the findings as moderate or low. We found that spontaneous vaginal birth was more likely to be associated with positive outcomes, followed by elective CS, and where women needed interventions, outcomes and experiences were generally better for AVD than for emergency CS. Where indicated, AVD can be an effective, acceptable alternative to caesarean section. Parents’ experience of AVD is improved by positive relationships, good communication, being involved in making decisions, and believing in the reason for AVD. Professionals’ attitudes and skills influence the use of AVD.

## Background

Assisted Vaginal Delivery (AVD) is a vaginal birth with the help of an instrument, usually forceps or vacuum. It is commonly performed for complications such as actual or imminent fetal compromise, to shorten the second stage of labour for maternal benefit, or for prolonged second stage of labour, especially where the fetal head is malrotated. AVD has the potential to improve maternal and newborn health and outcomes in any setting where the maternal and fetal condition require the rapid birth of the baby, and where it can be done safely. This may be particularly valuable in settings where caesarean section is not available, and where, even if available, surgical safety or safe management of complications cannot be guaranteed [1–3]. This is a particular issue when the woman is late in labour and the fetal head is very low in the pelvis.

Overuse of caesarean section has been a growing global concern during the last decades [4]. In 1985, the World Health Organization (WHO) stated that there was “no justification for any region to have a caesarean section rate higher than 10-15%” [5]. This was based on the scarce evidence available at that time. Since then, the rates of caesarean section have increased steadily in both HIC and LMIC countries [6]. This trend has not been accompanied by significant maternal or perinatal benefits; on the contrary, there is evidence that beyond a certain threshold, increasing caesarean section rates may be associated with increased maternal and perinatal morbidity. In low income settings particularly, the intrinsic risks associated with a surgical procedure such caesarean section also leave women and babies in a more vulnerable situation [1, 2, 7, 8]. In 2015, the WHO released a new Statement on Caesarean Section rates which superseded the earlier 1985 Statement emphasizing that “At population level, caesarean section rates higher than 10% are not associated with reductions in maternal and newborn mortality rates” and that “every effort should be made to provide caesarean sections to women in need, rather than striving to achieve a specific rate” [9, 10]. In October 2018, a new WHO guideline was released: WHO recommendations on non-clinical interventions to reduce unnecessary caesarean sections. Although the available evidence is limited, WHO includes recommendations on education and support for expectant mothers, implementation of clinical guidelines, audit and feedback, mandatory second opinion before conducting a caesarean section, models of childbirth care and financial disincentives for doctors and systems [11].

Although forceps and vacuum are not inherently dangerous, inappropriate decision making about when to use them, or sub-standard level of technical skills or training can cause iatrogenic harm, and this could disincentivize their use in favour of a caesarean section (if this is possible and a safe option locally) or even be a barrier to their use where they are the only technical solution available [2, 3]. The practice of AVD is more prevalent in high-income countries than in low- and middle-income settings [12]. A recent study of AVD use in 40 low- and middle-income countries found the most common reasons for not performing AVD were lack of equipment, lack of sufficiently trained staff, and national and institutional policies [12]. Other barriers may include misplaced perceptions that risk of mother to child HIV transmission is increased with use of AVD [3].

Given the potential benefits of AVD in terms of improving maternal and newborn health and outcomes and reducing caesarean section use, we aimed in this review to improve understanding of the limitations, barriers and potential facilitating factors for the appropriate use of AVD, from the point of view of women, service providers, policy makers, and funders. We therefore asked the following questions:

1. What views, beliefs, concerns and experiences have been reported in relation to AVD?
2. What are the influencing factors (barriers) associated with low use of/acceptance of AVD?
3. What are the enabling factors associated with increased appropriate use of/acceptance of AVD?

## Methods

A protocol for the review was published in the International Prospective Register of Systematic Reviews [13] prior to completion of the searches. We used a systematic sequential mixed-methods design [14]. The review was carried out according to the protocol with the following exceptions: no subgroup analyses were carried out due to insufficient data, and we decided by consensus to include PhD theses if they met the inclusion criteria and the data were not also reported in an associated publication.

### Criteria for study inclusion

Our focus was on the views, beliefs and experiences of maternity service users (including birth companions), health care providers, policy makers and funders regarding the acceptability, applicability and safety of, and knowledge and confidence in, AVD, which facilitate or inhibit its appropriate use. We included studies with qualitative designs (e.g. ethnography, phenomenology) or qualitative methods for data collection (e.g. focus group interviews, individual interviews, observation, diaries, oral histories), and studies using quantitative surveys and audits. There were no language restrictions. Studies from any country were eligible for inclusion; we defined low- and middle-income countries according to the OECD’s list of official development assistance recipients effective as at 1 January 2018. We limited our searches to studies published on or after 1985, the year of the first WHO statement on optimal caesarean section rates. Studies whose principal focus was breech presentation, multiple pregnancies, or those who have experienced a transverse or oblique lie or preterm birth were not included.

### Reflexive note

The authors varied in disciplinary backgrounds and experiences that may have influenced their input. In accordance with good practice in qualitative research [15] we considered our biases throughout the process and conferred regularly to reduce the impact on our findings. NC is health researcher whose research on breastfeeding and the postnatal period has informed her views on the importance of understanding and respecting women’s views and needs throughout the perinatal period. CK is a medical sociologist who held prior beliefs about mode of birth informed by interviews with women who have experienced primary assisted and spontaneous vaginal birth, planned and unplanned caesarean birth. MCB is a qualitative health researcher whose background has led her to focus on women’s voices in medical discourses. APB is a medical officer with over 15 years of experience in maternal and perinatal health research and public health. SD is a Professor of Midwifery; her interactions with the data were informed by her experience of supporting childbearing women as they experienced AVD. This included both brutal and disrespectful and sometimes unnecessary AVD that left women devastated, and careful, respectful AVD that left them joyful and positive. She strongly believes that respect for the physiology of birth and for women’s values and beliefs is the basis for understanding when and how to undertake AVD, and when and how to discuss this option with labouring women and partners.

### Search strategy

Systematic searches were carried out in April 2019 in CINAHL, MEDLINE, PsycINFO, EMBASE, Global Index Medicus, POPLINE, African Journals Online and LILACS. Searches were carried out using keywords for the Population, Intervention, and Outcomes where possible, or for smaller databases, using intervention keywords only. An example search strategy is shown in Additional File 1. In addition to systematic searches of electronic databases, we searched the reference lists of all included studies and the key references (i.e. relevant systematic reviews), both back chaining and forward checking for any references not identified in the electronic searches which may also be relevant. The following grey literature databases were searched: Open Grey, Open access thesis & dissertations, and Ethos.

### Study selection

Records were collated into Covidence systematic review software [16] and duplicates removed. Each abstract was independently assessed against the a priori inclusion/exclusion criteria by two review authors and irrelevant records discarded. Full texts of remaining papers were independently assessed by two review authors for eligibility, discrepancies adjudicated by a third reviewer, and the final list of included studies agreed among the reviewers.

### Data extraction and quality assessment

Study characteristics (details of the study, authors, study design, methods, intervention(s), population and results) were collected on a data extraction form. Quality of quantitative studies using a survey design was assessed using a critical appraisal checklist for a questionnaire study [17, 18], after which studies were graded A–D by discussion between two authors based on the outcome of the checklist. Quality of qualitative studies was assessed using the criteria from Walsh & Downe [19] and the A–D grading of Downe [20]. Initially, a pilot quality assessment of three studies was carried out by two authors independently to assess feasibility of the quality assessment tools. Then the studies were assessed by one, and checked by a second, review author. Disagreements were resolved through discussion, or by consulting a third review author.

### Data synthesis

Qualitative data was analysed using the principles of meta-ethnography [21]. The approach was comprised of five stages 1) Familiarisation and quality assessment; 2) Data extraction; 3) Coding; 4) Interpretative synthesis; and 5) CERQual assessment [22]. Two review authors (NC, CK), undertook coding and interpretive synthesis, with consensus reached in discussion with a third author (MCB). Starting with the earliest published paper [23], review authors read each study in detail, and independently extracted the results reported by the study authors, including any relevant verbatim quotes, along with the themes/theories/metaphors. Codes were constructed from the extracted data from the index paper and compared with data from each of the other papers until all the data had been coded into initial concepts. Data could be coded to more than one initial concept if this seemed appropriate. Initial concepts were discussed, refined and agreed by consensus before being coalesced into emergent themes. Themes were constructed by comparing similarities between the studies already analysed, and the one currently under review (‘reciprocal analysis’), and by looking for what might be different between the previous analysis and the paper currently under review (‘refutational analysis’). The emergent themes comprised the review findings. These were grouped into final themes and the resultant thematic structure was synthesised into a line of argument synthesis [21]. Degree of confidence which can be placed in each review finding was then assessed using the GRADE CERQual approach [22], in which each finding was assessed having either minor, moderate, or substantial concerns with respect to each of four domains: 1. methodological limitations of included studies; 2. relevance of the included studies to the review question; 3. coherence of the review finding; and 4. adequacy of the data contributing to a review finding. Then, based on an overall assessment of these four domains, confidence in the evidence for each review finding was assessed as high, moderate, low or very low.

Narrative synthesis of quantitative data from surveys and questionnaires was undertaken by two authors (SD, CK independently, with final decisions by consensus) [24]. Textual descriptions of individual studies were sub-grouped according to participants and factors of interest. Narrative summaries were then produced and organised thematically. There is currently no quantitative equivalent of CERQual for narrative summaries of survey data, but we agreed within our team that CERQual principles are transferable. We therefore applied CERQual criteria to the narrative summaries emerging from the survey and audit data. Finally, quantitative and qualitative data syntheses were combined using a ‘convergence coding matrix’. This approach illustrates the extent of *agreement, partial agreement, silence, or dissonance* between findings from included quantitative and qualitative studies [25]. The term *agreement* means that codes from more than one data set agree; *partial agreement* refers to agreement between some but not all data sets; *silence* refers to codes that are found in one data set but not others; and *dissonance* refers to disagreement between data sets, in meaning or salience.

## Results

**Fig. 1:**
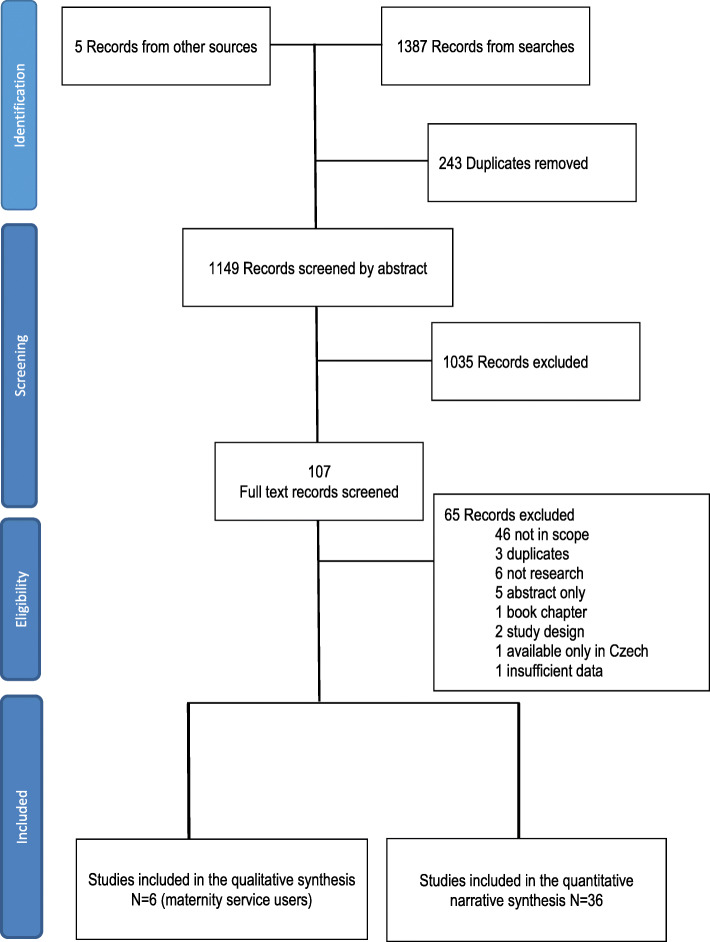
PRISMA diagram

From the searches, 1387 studies were identified, and a further five studies [23, 26–29] were identified from other sources. After 243 duplicates were removed, 1035 records were discarded as irrelevant after reviewing title and abstract. Of 107 full text papers screened, 65 records were excluded. This left 42 studies for quality assessment and synthesis [12, 23, 26, 28–66]. The earliest included studies were from 1985 [43, 45, 46] and the most recent from 2019 [29]. Figure 1 PRISMA Diagram illustrates the study selection process.

Six studies were qualitative studies of maternity service users reporting the views and experiences of 73 women and 20 men from three high-income countries (Sweden, UK, USA) [23, 26, 30–33]. The earliest study included in the qualitative evidence synthesis was from 2003 [23] and the most recent from 2015 [31]. It was not possible to conduct a qualitative evidence synthesis of provider data since only one (mixed-methods) study with qualitative data from healthcare providers was identified [34]. Four included survey studies [29, 36, 50, 66] reported some free-text responses. These papers, along with the six included qualitative studies [23, 26, 30–33] and the mixed-methods study [34] provided the starting point for our convergence coding matrix. In total 36 studies were included in the quantitative narrative synthesis, of which seven were from LMIC settings.

Table 1 gives an overview of the characteristics and quality assessment of all included studies. Thirty-five studies were from high-income countries, one from an upper-middle-income country, one from a lower middle-income country and three from least developed countries according to the OECD’s DAC list of Official Development Assistance Recipients 2018–2020. One study was a multi-country study of 40 LMICs and another was a multi-country survey. Thirty-one studies were rated A or B, and 11 rated C on quality assessment. No studies were excluded on grounds of quality. Fifteen of the 42 studies (36%) did not differentiate between forceps and ventouse, and of the quantitative surveys, in 33% (12/42), women and/or partners were asked about their experiences of AVD while on the postnatal ward [37, 38, 43, 44, 48, 49, 51, 52, 55, 56, 58, 61].

**Table 1:** Characteristics of included studies and quality assessment

Author & date	Resource setting	Country	Participants	Number of participants	Study Design	Methods	Quality Assessment
Alexander 2002 [34]	HIC	UK	Midwives	18	Mixed-method	Focus group and postal survey	**A**
Al-Mufti 1997 [35]	HIC	UK	Obstetricians	206	Quantitative	Postal survey	**C**
Avasarala 2009 [36]	HIC	UK	Postnatal mothers	58	Quantitative	Postal survey with free-text responses	**C**
Bailey 2017 [12]	LMIC	Up to 40 LMICs	Facility level data	Unclear	Quantitative	Descriptive secondary data analysis	**B**
Belanger-Levesque 2014 [37]	HIC	Canada	Postnatal mothers and fathers	400	Quantitative	In-patient survey	**B**
Chan 2002 [38]	HIC	UK	Postnatal mothers and fathers	226	Quantitative	In-patient survey	**B-**
Crosby 2017 [39]	HIC	Ireland, Canada	Obstetricians in training (qualified doctors registered as specialist trainees)	52	Quantitative	Online survey	**C**
Declercq 2008 [40]	HIC	USA	Postnatal mothers	1573	Quantitative	Telephone and on-line survey	**A-**
Fauveau 2006 [41]	LMIC	111 LMICs	Obstetricians, Midwives and Public Health specialists	Unclear	Quantitative	Face-to-face survey	**C-**
Fisher 1997 [42]	HIC	Australia	Primigravid women recruited during pregnancy with postnatal follow-up	272	Quantitative	Face-to-face survey	**B-**
Garcia 1985 [43]	HIC	UK	Postnatal mothers, Obstetricians, Paediatricians, and midwives	135	Quantitative	Face-to-face (women) and postal survey (staff)	**C**
^a^Goldbort 2009 [33]	HIC	USA	Postnatal women	10	Qualitative	Semi-structured interviews	**C**
Handelzalts 2017 [44]	HIC	Israel	Postnatal women	469	Quantitative	Self-complete survey	**C**
Healy 1985 [45]	HIC	USA, Canada	Obstetricians (Association Chairs and Training Programme Supervisors)	108	Quantitative	Postal survey	**B-**
Hildingsson 2013 [28]	HIC	Sweden	Primigravid and multiparous women recruited during pregnancy with postnatal follow-up	1763	Quantitative	Postal survey	**B**
Hewson 1985 [46]	HIC	Australia	Postnatal women	398	Quantitative	Face-to-face survey	**B-**
^a^Hurrell 2006 [26]	HIC	UK	Postnatal mothers and fathers	20	Qualitative	Semi-structured interviews	**A-**
Kjerulff 2018 [47]	HIC	USA	Primigravid women recruited during pregnancy with postnatal follow-up	3080	Quantitative	Face-to-face survey	**A**
Maaløe 2012 [66]	LMIC	Tanzania	Facility level data and eight staff (Nurse Midwives and Medical Officers)	152	Quantitative	Secondary data analysis and in-depth interviews	**A-**
Maclean 2000 [48]	HIC	England	Postnatal primiparous women	40	Quantitative	Postal survey	**C+**
Murphy 2003 [23]	HIC	UK	Postnatal women	27	Qualitative	Semi-structured interviews	**B**
Nolens 2018 [49]	LMIC	Uganda	Postnatal women	646	Quantitative	Face-to-face survey	**B**
Nolens 2019 [2, 29]	LMIC	Uganda	Postnatal women	759	Quantitative	Face-to-face survey with open responses	**B+**
Nystedt 2006 [32]	HIC	Sweden	Primiparous women	10	Qualitative	Semi-structured interviews	**B**
Ramphul 2012 [50]	HIC	UK& Ireland	Obstetricians (Labour ward leads and specialist trainees)	323	Quantitative	Postal survey	**A-**
Ranta 1995 [51]	HIC	Finland	Primigravid and multiparous women recruited during pregnancy with postnatal follow-up	1091	Quantitative	Self-complete survey and secondary data	**C**
Renner 2007 [52]	HIC	USA	Postnatal women	80	Quantitative	Self-complete survey	**B+**
Rijnders 2008 [53]	HIC	Netherlands	Postnatal women	1309	Quantitative	Postal survey	**B**
Rowlands 2012 [54]	HIC	England	Postnatal women	5332	Quantitative	Secondary analysis of national postal survey	**B**
Ryding 1998 [55]	HIC	Sweden	Postnatal women	326	Quantitative	Postal questionnaires	**B**
Salmon 1992 [56]	HIC	England	Primigravid women recruited during pregnancy with postnatal follow-up	110	Quantitative	Self-complete survey and secondary data	**C**
Sánchez Del Hierro 2014 [57]	LMIC	Ecuador	Medical graduates	90	Quantitative	Online survey	**A**
Schwappach 2004 [58]	HIC	Switzerland	Postnatal women	2079	Quantitative	Self-complete survey and secondary data	**A**
Shaaban 2012 [59]	LMIC	Egypt	Obstetricians (Consultants, specialists, registrars)	167	Quantitative	Self-complete survey	**B-**
Shorten 2012 [60]	HIC	Australia	Postnatal women	165	Quantitative	Self-complete survey	**B**
^a^Sjodin 2018 [30]	HIC	Sweden	Postnatal women	16	Qualitative	Semi-structured interviews	**B**
Uotila 2005 [61]	HIC	Finland	Postnatal women	205	Quantitative	Self-complete survey	**B**
Waldenström 1999 [62]	HIC	Sweden	Primigravid and multiparous women recruited during pregnancy with postnatal follow-up	1111	Quantitative	Self-complete survey	**A-**
Wiklund 2008 [63]	HIC	Sweden	Primigravid women recruited during pregnancy with postnatal follow-up	496	Quantitative	Self-complete survey	**C**
Wilson 2002 [64]	HIC	UK	Facility level data and five staff (Medical Director/Senior Obstetrician, Manager, Paediatrician, Midwife and middle-grade Obstetrician) from each of 20 hospitals	1100	Quantitative	Secondary data analysis and structured interview	**A**
Wright 2001 [65]	HIC	UK	Obstetricians in training (qualified doctors registered as specialist trainees)	279	Quantitative	Postal questionnaire	**A-**
Zwedberg 2015 [31]	HIC	Sweden	Postnatal fathers	10	Qualitative	Semi-structured interviews	**B**

^a^Three PhDs were identified; two of which had published papers. These two papers and the third PhD (unpublished) were included

From 36 included studies with quantitative data [12, 28, 29, 34–66], we derived eight narrative summaries, which we grouped into four thematic headings: prevalence of AVD use in practice; skills and attitudes (including professional and personal attitudes of healthcare professionals); experiences of the birth; and impact and consequences of AVD for women and partners. Table 2 shows the summary of quantitative review findings and associated confidence assessments. From the six included qualitative studies, [23, 26, 30–33], we derived 10 review findings, which mapped to four distinct final themes: ‘coming to know AVD by experience’, ‘turbulent feelings about the actual experience’, ‘trust, control, and relationships’, and ‘implications for future reproductive choices’. A summary of the initial concepts, emergent themes and final themes is shown in Table 3, while Table 4 shows the summary of review findings and associated CERQual assessment. Inevitable differences were apparent between the in-depth views and experiences framing of the qualitative studies and the structured preferences, opinions and outcomes framing of most of the quantitative studies. There was, however, agreement or partial agreement, evident across study designs, that the impact of unmet expectations/of unexpected events, good communication, and (believing in) the reason for intervention are all critical mediators of how actual birth experiences are perceived by women. Table 5 Convergence coding matrix shows triangulation of the qualitative and quantitative evidence synthesis and provides the structure for the reporting of findings hereafter. Summary of findings statements are highlighted in bold.

**Table 2:** Quantitative Summary of Findings and CERQual Assessment

Summary of findings	Studies	Type of mode of birth included	Comments	Confidence in this finding
**Prevalence of assisted vaginal delivery** Included studies indicate low levels of use of instrumental birth, and early default to CS. Lack of equipment and lack of trained staff contribute to this situation. Improved access to the Cochrane database was associated with an increased use of ventouse vs forceps over time in one UK study, but this was not explained by changes in individual staff knowledge attitudes, or access to Cochrane reviews.	Bailey 2017 [12] 40 LMIC countries [B]	vacuum, forceps, spontaneous	Two of the twelve surveys undertaken more than 30 years ago. Most studies of moderate or low quality. LMIC countries included and relatively recent. Most studies identify the instruments included	***Moderate*** Downgraded for study quality
	Crosby 2017 [39] Ireland Canada [C]	forceps		
	Fauveau 2006 [41] worldwide [C]	vacuum		
	Healy 1985 [45] US [B]	forceps		
	Hewson 1985 [46] Australia [B-]	forceps		
	Maaloe 2012 [66] Tanzania [A-]	vacuum, CS		
	Ramphul 2012 [50] UK [A]	AVD		
	Rowlands 2012 [54] UK [B]	forceps, spontaneous, elective and emergency CS		
	Ryding 1998 [55] Sweden [B]	AVD, spontaneous, elective and emergency CS		
	Schwappach 2004 [58] Switzerland [A]	AVD, spontaneous, emergency and elective CS		
	Uotila 2005 [61] Finland [B	vacuum		
	Wilson 2002 [64] UK [A]	vacuum, forceps		
**Skills (development) in assisted vaginal delivery** Mixed findings about the self-reported skills of obstetricians in determining the need for, seeking a second opinion in, and accuracy of clinical stills for, instrumental delivery. Evidence from one study that more junior doctors report being more likely to default to a CS, and that senior doctors are more aware than junior doctors that they make errors in some relevant clinical judgements. Less than 15% of responding LMICs in one multi-country audit reported teaching in AVD, as reported in 2006. In another survey most trainees report correct techniques for assessment prior to instrumental vaginal birth, but that, in practice, this is more difficult where women have insufficient pain relief, or where there is significant fetal caput, or where the practitioner is relatively inexperienced. In one study, Irish trainees were more likely to use AVD than Candian trainees, but confidence in AVD usedid not differ between the two groups. Midwives who were trained in using ventouse in the UK seemed to be confident in its use. Actual skills and competence were not tested in any included studies.	Alexander 2002 [34] UK [A]	vacuum	One of the seven surveys undertaken more than 30 years ago. Mix of high and low quality studies. Varying results across studies. Four UK. All but one study identify the instruments included	***Low*** Downgraded for study quality and coherence
	Crosby 2017 [39] Ireland, Canada [C]	forceps		
	Fauveau 2006 [41] worldwide [C]	vacuum		
	Garcia 1985 [43] UK [C]	forceps		
	Ramphul 2012 [50] UK [A]	AVD		
	Sanchez del Hierro 2014 [57] Equador [A-]	forceps		
	Wilson 2002 [64] UK [A]	forceps		
**Professional attitudes to the use of assisted vaginal delivery** In one US study undertaken in 1985, the attitude of the director of the obstetric training programme was not associated with the rate of forceps performed in their institution. One UK study showed that staff attitude was not a key determinant of a rise in use of ventouse over time. In an Egyptian study, nearly half of all obstetricians attending a conference rejected the use of instrumental birth (49%) with more experienced medical staff being more positive to AVD than more junior staff, and those working in the private sector less positive than those working in the public sector (check with full text. A survey of practitioners in 121 LMICs reported in 2006 indicated that practitioners in about half (48%) of the countries represented reported knowledge, positive attitude, teaching and countrywide use of the method,; 15% reported no knowledge and therefore no use in their country. Irish trainees were more likely to use AVD and were more comfortable with its use than Canadian trainees in one study.	Crosby 2017 [39] Ireland, Canada [C]	forceps	One of the six surveys undertaken more than 30 years ago. Most low or moderate quality. LMIC countries included and relatively recent. Varing results across the studies. All but one study identify the instruments used	***Low*** Downgraded for study quality and coherence
	Fauveau 2006 [41] worldwide [C]	vacuum		
	Healy 1985 [45] US [B]	forceps		
	Sanchez del Hierro 2014 [57] Equador [A-]	forceps		
	Shaaban 2012 [59] Egypt [B-]	AVD		
	Wilson 2002 [64] UK [A]	forceps		
**Personal attitudes to mode of birth for oneself/a partner** (obstetricians) Preference for elective CS amongst UK obstetricians (for them/their partners) was around 16% (15–17%) in both 1997 and 2001. A majority in both time periods would be happy to have an instrumental birth as an alternative for mid-cavity arrest, especially if they could choose the operator. Junior staff in 1997 were more likely than senior staff to choose ventouse than forceps for arrested labour, for both OP and OA positions. Choices were not affected by gender, age, or hospital status.	Al-Mufti 1997 [35] UK [C]	forceps, spontaneous, elective CS	One of the two studies undertaken more than 20 years ag, but this is not a limitation in this case as one of the aims is historical comparison. Both studies from the UK, quality from high to low, instruments not identified in one.	***Very low*** downgraded for relevance, quality and adequacy
	Wright 2001 [65] UK [A-]	AVD, spontaneous, elective CS		
**Women’s experiences of assisted vaginal delivery**. In all studies where spontaneous physiological birth is included, it scores the highest for a positive experience. In some, elective CS scores almost as highly. Having an unplanned mode of birth (emergency CS or instrumental, especially with an episiotomy, and especially where the intervention is done for delay in labour rather than for acute clinical risk) seems to be associated with less positive reports of childbirth experience for women. In some studies, emergency CS is rated as the least positive of all birth modes, followed by instrumental, with a better experience reported after ventouse than forceps in most, but not all comparisons. In others, instrumental birth with episiotomy is the most distressing, especially after a ToL following a previous CS. A few studies note that negative experience is associated with poor pain relief, but in one study women with AVD reported higher levels of pain relief than women with spontaneous birth Where longer term memories of birth experience are recorded, the differences reported immediately after birth persist (up to 3 years in one study).	Avasarala 2009 [36] UK [C]	AVD, CS	Five of the 16 surveys undertaken more than 20 years ago. Most of low or moderate quality. Only one in a low income country. Instruments not identified in seven of the 16 studies	***Low*** Downgraded for study quality and relevance
	Garcia 1985 [43] UK [C]	forceps		
	Handelzalts 2017 [44] US [C]	spontaneous, emergency and elective CS		
	Hewson 1985 [46] Australia [B-]	forceps		
	Hildingsson 2013 [28] Sweden [B]	AVD, spontaneous		
	Kjerulff 2018 [47] USA A-	CS, AVD		
	Maclean 2000 [48] UK [C+]	spontaneous, forceps, emergency CS		
	Nolens 2019 [49] Uganda [B+]	CS		
	Ranta 1995 [51] Finland [C]	vacuum,‘, urgent’ and emergency CS		
	Rijnders 2008 [53] Netherlands [B]	AVD home, (spontaneous), emergency CS		
	Salmon 1992 [56] UK [C]	forceps, spontaneous, CS		
	Schwappach 2004 [58] Switzerland [A]	AVD, spontaneous, emergency and elective CS		
	Shorten 2012 [60] USA [B]	AVD, spontaneous, emergency and elective CS		
	Uotila 2005 [61] Finland [B]	vacuum		
	Waldenstrom 1999 [62] Sweden [A-]	spontaneous, vacuum, CS		
	Wiklund 2008 Sweden [C]	AVD, spontaneous, emergency and elective CS		
**Communication, information and consent** Some evidence that many women do not have information about the risks and benefits of AVD (plus or minus episiotomy), either antenatally, intrapartum when the procedure is used, or postnatally to explain what happened.	Avasarala 2009 [36] UK [C]	AVD, CS	One of the six surveys undertaken more than 30 years ago. All of low or moderate quality. Instruments not identified in three studies	***Moderate*** Downgraded for study quality
	Fauveau 2006 [41] worldwide [C]	vacuum		
	Garcia 1985 [43] UK [C]	forceps		
	Ramphul 2012 [50] UK [A]	AVD		
	Renner 2007 [52] USA [C]	AVD, elective CS		
	Uotila 2005 [61] Finland [B]	vacuum		
**Impact of assisted vaginal delivery (women)** Studies have variously measured postnatal mood, sexual function, desire to have more children, dyspareunia, urinary and bowel problems, postnatal fear of childbirth, pain, haemorrhoids, and backache, Having a spontaneous vaginal birth without instruments or episiotomy seems to result in the most positive outcomes in the short and longer term (though this is not the case for a few variables). Having an unplanned mode of birth may be the strongest predictor of negative outcomes. In some studies, emergency CS is associated with least positive impacts, followed by instrumental (negative outcomes reported for both forceps or ventouse in some studies – others show better outcomes for ventouse than CS in the short and longer term). In others, instrumental birth is the most distressing. Surveys that assessed preference for mode of birth next time indicate that spontaneous vaginal delivery is preferred by most, with some preferring a planned CS, and most preferring instrumental birth over emergency CS. If an instrumental birth is required, most seem to prefer ventouse over forceps.	Avasarala 2009 [36] UK [C]	AVD, CS	Three of the 14 papers report studies undertaken more than 20 years ago. Most of low or moderate quality. Two in the same LMIC setting, over the same time period. Instruments not identified in seven studies	***Low*** Downgraded for study quality and relevance
	Chan 2002 [38] UK [B]	AVD, spontaneous, CS		
	Declercq 2008 [40] USA [A]	AVD, spontaneous, CS		
	Fisher 1997 [42] Australia [B+]	forceps, spontaneous, CS		
	Garcia 1985 [43] UK [C]	forceps		
	Handelzalts 2017 US [C]	spontaneous, emergency and elective CS		
	Hildingsson 2013 [28] Sweden [B]	AVD, spontaneous		
	Nolens 2019 [2, 29] Uganda [B+]	vacuum, CS		
	Nolens 2018 [49] Uganda [B+]	vacuum, CS		
	Rowlands 2012 [54] UK [B]	forceps, spontaneous, elective and emergency CS		
	Ryding 1998 [55] Sweden [B]	AVD, spontaneous, elective and emergency CS		
	Schwappach 2004 [58] Switzerland [A]	AVD, spontaneous, emergency and elective CS		
	Uotila 2005 [61] Finland [B]	vacuum		
	Wiklund 2008 Sweden [C]	AVD spontaneous, emergency and elective CS		
**Experience of witnessing assisted vaginal delivery (partners)** Witnessing an emergency CS or instrumental birth seems to be associated with less positive reports of childbirth for partners than a spontaneous vaginal birth. Emergency CS seems to be associated with marginally higher scores than instrumental birth, but only two studies measure this comparison. In one study, partners reported having panic attacks during the birth, and a few said they wouldn’t have more children. Some would prefer their partner chose an elective cs next time.	Belanger-Levesque 2014 [37] Canada [B]	AVD, spontaneous, elective and emergency CS	All three included studies relatively recent. All of moderate quality. None in an LMIC setting. Instruments not identified in any of the included studies	***Low*** Downgraded for quality and relevance
	Chan 2002 [38] UK [B]	AVD, spontaneous, CS		
	Hildingsson 2013 [28] Sweden [B]	AVD, spontaneous

**Table 3:** Qualitative evidence synthesis: summary of initial concepts, emergent themes and final themes

Initial concepts	Emergent themes/SoFs	Studies contributing to review finding	Final themes	Line of argument synthesis
Operative delivery not contemplated	**Expectations and preparedness for AVD - a birth you couldn’t plan for**	Hurrell 2006 [26]	**Coming to know AVD by experience**	In high income settings, it might be inevitable that women will be unprepared for an AVD because it is not an outcome readily considered: women may not be offered, or may avoid, antenatal education, and it is an outcome arising from an unexpected chain of events making it difficult to prepare for. Because of this, women’s condition, adequate pain relief and interactions with staff are all the more important. Assisted vaginal delivery is an intervention that can be frightening and invasive; it can be experienced as violent. Women can feel like failures, and women and partners can also feel relief and positive emotions. Women and partners may need to understand why an AVD was the right care for them (indication). Views on future delivery mode are mixed including increased confidence for a vaginal birth and preferences for a future caesarean birth.
		Murphy 2003 [23]		
Births plans meaningless				
Antenatal education				
Keeping an open mind				
Perception of necessity	**Beliefs about need/indications for AVD**	Hurrell 2006 [26]		
Feelings of failure		Murphy 2003 [23]		
Beliefs about problems with baby				
Unable to recall				
Finding a context for their birth experience	**Reconciling/coping with personal experience**	Hurrell 2006 [26]		
Difficulties with moving on				
Effective pain relief absence of major concern with AVD	**Pain during assisted vaginal delivery**	Hurrell 2006 [26]	**Turbulent feelings about the actual experience**	
		Sjödin 2018 [30]		
Working with pain/enabler		Nystedt 2006 [32]		
Experiencing pain as traumatic (barrier)				
		Zwedberg 2015 [31]		
Violence and injury	**Frightening and violent experiences**	Hurrell 2006 [26]		
Being possessed by fear and distress		Sjödin 2018 [30]		
Being conscious, but somewhere else		Nystedt 2006 [32]		
		Zwedberg 2015 [31]		
		Goldbort 2009 [33]		
Fathers feeling positive and emotional	**Positive or beneficial reactions**	Hurrell 2006 [26]		
		Zwedberg 2015 [31]		
Fathers coping strategies – finding strength to support their partners		Nystedt 2006 [32]		
Relief of an end to labour				
Feeling unperturbed				
To be part of a team	**Active participation through collaboration and involvement**	Hurrell 2006 [26]	**Trust, control and relationships**	
Wish to be involved in decision-making		Zwedberg 2015 [31]		
Fathers feelings of inclusion/exclusion		Sjödin 2018 [30]		
Lack of trust in caregiver	**Balancing control and trust**	Hurrell 2006 [26]		
Balancing feelings of control and trust		Zwedberg 2015 [31]		
Feeling of loss of control		Sjödin 2018 [30]		
		Nystedt 2006 [32]		
		Goldbort 2009 [33]		
Communication	**The need to understand and be understood**	Hurrell 2006 [26]		
To understand		Zwedberg 2015 [31]		
		Sjödin 2018 [30]		
Put off a future pregnancy	**Mixed views about any future pregnancy and delivery**	Hurrell 2006 [26]	**Implications for future reproductive choices**	
More confident about a future vaginal delivery		Murphy 2003 [23]		
Preference for a caesarean		Zwedberg 2015 [31]

**Table 4:** CERQual Summary of findings (SoFs)

Review finding	Studies contributing to review finding	CERQual Assessment	Explanation of confidence in the evidence assessment
**Coming to know AVD by experience**			
**Expectations and preparedness for AVD - a birth you couldn’t plan for** Women and men reported views of assisted vaginal deliveries as a birth experience that you couldn’t plan for. In some cases, this was because an assisted vaginal delivery had simply not been contemplated, with women’s birth preparations focused elsewhere. While women perceived an absence of information about forceps or ventouse, compared to spontaneous vaginal birth or caesarean section, there was an appreciation of the difficulties surrounding information about assisted vaginal delivery, which not everyone needs to know, and not everyone desires to know. Although assisted vaginal delivery was reported to be a missing component of antenatal preparation, other parents described their own self-imposed limitations on preparation.	Murphy 2003 [23]	Low confidence	Major concerns regarding adequacy (two studies from one country). Moderate concerns regarding coherence.
	Hurrell 2006 [26]		
**Beliefs about need/indications for AVD** Some parents described an acceptance of assisted vaginal delivery based on their perception of necessity. In some cases, there was a lack of understanding about what happened, when and why. Some women understood that there had been a problem with either themselves or their baby, which some women viewed as a failure on their part to deliver vaginally. Some women could not remember any explanation from a health professional as to what happened, others could remember being spoken to, but not what it was about.	Murphy 2003 [23]	Low confidence	Major concerns regarding adequacy (two studies from one country). Moderate concerns regarding coherence.
	Hurrell 2006 [26]		
**Reconciling/coping with experience -** Women described finding a context for their birth experience that allowed them to come to terms with it. Conversely some women had difficulties with moving on, describing feels of low mood and low self-worth.	Hurrell 2006 [26]	Low confidence	Major concerns regarding adequacy (only one study). Moderate concerns regarding coherence.
**Turbulent feelings about the actual experience**			
**Pain-** For some women, effective pain relief allowed an absence of major concerns about the procedure, and for other women who did experience pain, compassionate support enabled them to work with it. However, some women experienced pain as traumatic (self-reported), and men expressed concerns that their partners would be traumatized too (as witnessed by partner).	Hurrell 2006 [26]	Moderate confidence	Major concerns about adequacy (studies from only two countries).
	Nystedt 2006 [32]		
	Zwedberg 2015 [31] Sjödin 2018 [30]		
**Frightening and violent experience** - Some women and men experience AVD as frightening, distressing or violent. Participants use vivid language to describe the sights and sounds of their experience - seeing blood, perceptions of force or violence (words like tearing, ripping, dragging), the baby’s appearance afterward. Participants described the emotional impact of the experience in terms of fear or distress and a few participants relate experiences of dissociation or trying to avoid perceiving/experiencing anything.	Hurrell 2006 [26]	Moderate confidence	Moderate concerns about adequacy (studies from three countries).
	Nystedt 2006 [32]		
	Goldbort 2009 [33]		
	Zwedberg 2015 [31]		
	Sjödin 2018 [30]		
**Beneficial or positive reactions** - Women and men reported a range of positive reactions after experiencing an AVD. These included feeling unperturbed by having an AVD, to feeling relief that labour is over, to feelings of joy at the birth of the baby. Men described finding strength to cope with a difficult situation to support their partners.	Hurrell 2006 [26]	Moderate confidence	Major concerns about adequacy (studies from only two countries).
	Zwedberg 2015 [31]		
	Nystedt 2006 [32]		
**Barriers and facilitators**			
**Trust, control and relationships**			
**Active participation through collaboration and involvement** - Both women and men wished to feel part of a team with care providers and to be involved in decision making. Men expressed feelings of being excluded, but wishing to be involved.	Hurrell 2006 [26]	Moderate confidence	Major concerns about adequacy (studies from only two countries).
	Sjödin 2018 [30] Zwedberg 2015 [31]		
**Balancing control and trust -** The amount of trust that women and men have in their care givers at the time of an assisted vaginal delivery is linked both to their perceptions of control and to their acceptance of the intervention.	Hurrell 2006 [26]	Moderate confidence	Moderate concerns about adequacy (studies from three countries).
	Nystedt 2006 [32]		
	Goldbort 2009 [33]		
	Zwedberg 2015 [31]		
	Sjödin 2018 [30]		
**The need to understand and to be understood -** The quality of communication between caregivers, women and men at the time of an assisted vaginal delivery was key. Women appreciated care in what was said and how it was said. They wanted information and to be listened to as a means to retaining some degree of involvement in something they had little control over.	Hurrell 2006 [26]	Moderate confidence	Major concerns about adequacy (studies from only two countries).
	Zwedberg 2015 [31]		
	Sjödin 2018 [30]		
**Implications for future reproductive choices**			
**Mixed views about any future pregnancy and delivery** - AVD impacts on women and men views about future pregnancies - In some cases, the experience of an assisted vaginal delivery put women off planning another pregnancy, while for other women and some men, it meant that they had stronger views about a particular birth mode. Some women, and men, described preferring a caesarean for any future birth. Other women, and men, felt better prepared for labour and a future vaginal delivery.	Murphy 2003 [23]	Moderate confidence	Major concerns about adequacy (studies from only two countries).
	Hurrell 2006 [26]		
	Zwedberg 2015 [31]

**Table 5:** Triangulation of qualitative evidence synthesis and quantitative narrative synthesis at summary of findings level

	Qualitative evidence synthesis		Convergence coding matrix				Quantitative narrative synthesis		
**Barriers and facilitators to AVD**	**Summary of findings**	**Studies**	**Agreement**	**Partial agreement**	**Silence**	**Dissonance**	**Studies**	**Summary of findings**	
							**Prevalence of AVD use in practice**		
		0 studies			✓		Bailey 2017 [12]	**Prevalence of assisted vaginal delivery (Moderate confidence)** Included studies indicate low levels of use of instrumental birth, and early default to CS. Lack of equipment and lack of trained staff contribute to this situation. Improved access to the Cochrane database was associated with an increased use of ventouse vs forceps over time in one UK study, but this was not explained by changes in individual staff knowledge attitudes, or access to Cochrane reviews.	**Views of AVD use in practice**
							Crosby 2017 [39]		
							Fauveau 2006 [41]		
							Healy 1985 [45]		
							Hewson 1985 [46]		
							Maaloe 2012 [66]		
							Ramphul 2012 [50]		
							Rowlands		
							Ryding 1998 [55] Schwappach 2004 [58]		
							Uotila 2005 [61]		
							Wilson 2002 [64]		
		0 Studies			✓		Alexander 2002 [34]	**Skills (development) in assisted vaginal delivery (Low confidence)** Mixed findings about the self-reported skills of obstetricians in determining the need for, seeking a second opinion in, and accuracy of clinical stills for, instrumental delivery. Evidence from one study that more junior doctors report being more likely to default to a CS, and that senior doctors are more aware than junior doctors that they make errors in some relevant clinical judgements. Less than 15% of responding LMICs in one multi-country study reported teaching in AVD, as reported in 2006. In another survey most trainees report correct techniques for assessment prior to instrumental vaginal birth, but that, in practice, this is more difficult where women have insufficient pain relief, or where there is significant fetal caput, or where the practitioner is relatively inexperienced. In one study, Irish trainees were more likely to use AVD than Canadian trainees, but confidence in AVD use did not differ between the two groups. Midwives who were trained in using ventouse in the UK seemed to be confident in its use. Actual skills and competence were not tested in any included studies..	
							Crosby 2017 [39]		
							Fauveau 2006 [41]		
							Garcia 1985 [43] Ramphul Sanchez del Hierro 2014 [57]		
							Wilson 2002 [64]		
							**Skills and attitudes**		
		0 studies			✓		Crosby 2017 [39]	**Professional attitudes to the use of assisted vaginal delivery (Low confidence)** In one US study undertaken in 1985, the attitude of the director of the obstetric training program was not associated with the rate of forceps performed in their institution. One UK study showed that staff attitude was not a key determinant of a rise in use of ventouse over time. In an Egyptian study, nearly half of all obstetricians attending a conference rejected the use of instrumental birth (49%) with more experienced medical staff being more positive to AVD than more junior staff, and those working in the private sector less positive than those working in the public sector (check with full text. A survey of practitioners in 121 LMICs reported in 2006 indicated that practitioners in about half (48%) of the countries represented reported knowledge, positive attitude, teaching and countrywide use of the method; 15% reported no knowledge and therefore no use in their country. Irish trainees were more likely to use AVD and were more comfortable with its use than Canadian trainees in one study.	
							Fauveau 2006 [41]		
							Healy 1985 [45]		
							Sanchez del Hierro 2014 [57]		
							Shaaban 2012 [59]		
							Wilson 2002 [64]		
		0 studies			✓		Al-Mufti 1997 [35]	**Personal attitudes to mode of birth for oneself/a partner (obstetricians) (Very low confidence)** Preference for elective CS amongst UK obstetricians (for them/their partners) was around 16% (15–17%) in both 1997 and 2001. A majority in both time periods would be happy to have an instrumental birth as an alternative for mid-cavity arrest, especially if they could choose the operator. Junior staff in 1997 were more likely than senior staff to choose ventouse than forceps for arrested labour, for both OP and OA positions. Choices were not affected by gender, age, or hospital status.	**Experiences AVD**
							Wright 2001 [65]		
	**Coming to know AVD by experience**						**Experience of the birth**		
	**Expectations and preparedness for assisted vaginal delivery - a birth you couldn’t plan for (Low confidence)** Women and men reported views of assisted vaginal deliveries as a birth experience that you couldn’t plan for. In some cases, this was because an assisted vaginal delivery had simply not been contemplated, with women’s birth preparations focused elsewhere. While women perceived an absence of information about forceps or ventouse, compared to spontaneous vaginal birth or caesarean section, there was an appreciation of the difficulties surrounding information about assisted vaginal delivery, which not everyone needs to know, and not everyone desires to know. Although assisted vaginal delivery was reported to be a missing component of antenatal preparation, other parents described their own self-imposed limitations on preparation.	Hurrell 2006 [26] Murphy 2003 [23]		✓			Avasarala 2009 [36]	**Women’s experiences of assisted vaginal delivery (Low confidence)** In all studies where spontaneous physiological birth is included, it scores the highest for a positive experience. In some, elective CS scores almost as highly. Having an unplanned mode of birth (emergency CS or instrumental, especially with an episiotomy, and especially where the intervention is done for delay in labour rather than for acute clinical risk) seems to be associated with less positive reports of childbirth experience for women. In some studies, emergency CS is rated as the least positive of all birth modes, followed by instrumental, with a better experience reported after ventouse than forceps in most, but not all comparisons. In others, instrumental birth with episiotomy is the most distressing, especially after a trial of labour following a previous CS. A few studies note that negative experience is associated with poor pain relief, but in one study women with AVD reported higher levels of pain relief than women with spontaneous birth. Where longer term memories of birth experience are recorded, the differences reported immediately after birth persist (up to 3 years in one study).	
							Garcia 1985 [43]		
							Handelzalts 2017 [44]		
							Hewson 1985 [46]		
							Hildingsson 2013 [28]		
							Kjerulff 2018 [47] Maclean 2000 [48] Nolens 2019 [2, 29]		
							Ranta 1995 [51]		
	**Beliefs about need/indications for assisted vaginal delivery (Low confidence)** Some parents described an acceptance of assisted vaginal delivery based on their perception of necessity. In some cases, there was a lack of understanding about what happened, when and why. Some women understood that there had been a problem with either themselves or their baby, which some women viewed as a failure on their part to deliver vaginally. Some women could not remember any explanation from a health professional as to what happened, others could remember being spoken to, but not what it was about.	Hurrell 2006 [26] Murphy 2003 [23]	✓						
							Rijnders 2008 [53]		
							Salmon 1992 [56] Schwappach 2004 [58] Shorten 2012 [60] Uotila 2005 [61] Waldenstrom 1999 [62]		
	**Reconciling/coping with personal experience of assisted vaginal delivery (Low confidence)** Women described finding a context for their birth experience that allowed them to come to terms with it. Conversely some women had difficulties with moving on, describing feels of low mood and low self-worth.	Hurrell 2006 [26]		✓					
							Wiklund 2008 [63]		
	**Turbulent feelings about the actual experience**								
	**Pain during assisted vaginal delivery (Moderate confidence)** For some women, effective pain relief allowed an absence of major concerns about the procedure, and for other women who did experience pain, compassionate support enabled them to work with it. However, some women experienced pain as traumatic and men expressed concerns that their partners would be traumatised.	Hurrell 2006 [26] Sjödin 2018 [30] Nystedt 2006 [32] Zwedberg 2015 [31]	✓						
	**Frightening and violent experiences during assisted vaginal delivery (Moderate confidence)** Some women and men experience AVD as frightening, distressing or violent. Participants use vivid language to describe the sights and sounds of their experience – seeing blood, perceptions of force or violence (words like tearing, ripping, dragging), the baby’s appearance afterward. Participants described the emotional impact of the experience in terms of fear or distress and a few participants relate experiences of dissociation or trying to avoid perceiving/experiencing anything.	Hurrell 2006 [26] Sjödin 2018 [30] Nystedt 2006 [32] Zwedberg 2015 [31] Goldbort 2009 [33]	✓						
	**Positive or beneficial reactions during assisted vaginal delivery (Moderate confidence)** Women and men reported a range of positive reactions after experiencing an AVD. These included feeling unperturbed by having an AVD, to feeling relief that labour is over, to feelings of joy at the birth of the baby. Men described finding strength to cope with a difficult situation to support their partners.	Hurrell 2006 [26] Zwedberg 2015 [31] Nystedt 2006 [32]	✓						
	**Trust, control and relationships**								**Living after experiences of AVD**
	**Active participation through collaboration and involvement (Moderate confidence)** Both women and men wished to feel part of a team with care providers and to be involved in decision making. Men expressed feelings of being excluded but wishing to be involved.	Hurrell 2006 [26] Zwedberg 2015 [31] Sjödin 2018 [30]		✓			Avasarala 2009 [36] Fauveau 2006 [41] Garcia 1985 [43] Ramphul 2012 [50] Renner 2007 [52]	**Communication, information and consent (Moderate confidence)** Some evidence that many women do not have information about the risks and benefits of AVD (plus or minus episiotomy), either antenatally, intrapartum when the procedure is used, or postnatally to explain what happened.	
	**Balancing control and trust (Moderate confidence)** The amount of trust that women and men have in their caregivers at the time of an assisted vaginal delivery is linked both to their perceptions of control and to their acceptance of the intervention.	Hurrell 2006 [26], Zwedberg 2015 [31] Sjödin 2018 [30] Nystedt 2006 [32] Goldbort 2009 [33]		✓					
							Uotila 2005 [61]		
	**The need to understand and be understood (Moderate confidence)** The quality of communication between caregivers, women and men at the time of an assisted vaginal delivery was key. Women appreciated care in what was said and how it was said. They wanted information and to be listened to as a means to retaining some degree of involvement in something they had little control over.	Hurrell 2006 [26] Zwedberg 2015 [31] Sjödin 2018 [30]		✓					
	**Implications of AVD for future reproductive choices**						**Impact and consequences of AVD for women and partners**		
	**Mixed views about any future pregnancy and delivery (moderate confidence)** AVD impacts on women and men views about future pregnancies - In some cases, the experience of an assisted vaginal delivery put women off planning another pregnancy, while for other women and some men, it meant that they had stronger views about a particular birth mode. Some women, and men, described preferring a caesarean for any future birth. Other women, and men, felt bettepared for labour and a future vaal delivery.	Hurrell 2006 [26] Murphy 2003 [23] Zwedberg 2015 [31]	✓				Avasarala 2009 [36] Chan 2002 [38]	**Impact of assisted vaginal delivery (women) (Low confidence)** Studies have variously measured postnatal mood, sexual function, desire to have more children, dyspareunia, urinary and bowel problems, postnatal fear of childbirth, pain, haemorrhoids, and backache, Having a spontaneous vaginal birth without instruments or episiotomy seems to result in the most positive outcomes in the short and longer term (though this is not the case for a few variables). Having an unplanned mode of birth may be the strongest predictor of negative outcomes. In some studies, emergency CS is associated with least positive impacts, followed by instrumental (negative outcomes reported for both forceps or ventouse in some studies – others show better outcomes for ventouse than CS in the short and longer term). In others, instrumental birth is the most distressing. Surveys that assessed preference for mode of birth next time indicate that spontaneous VD is preferred by most, with some preferring a planned CS, and most preferring instrumental birth over emergency CS. If an instrumental birth is required, most seem to prefer ventouse over forceps.	
							Declercq 2008 [40]		
							Fisher 1997 [42]		
							Garcia 1985 [43] Handelzalts 2017 [44] Hildingsson 2013 [28]		
							Nolens 2019 [2, 29]		
							Nolens 2018 [49] Rowlands 2012 [54] Ryding 1998 [55]		
							Schwappach 2004 [58]		
							Uotila 2005 [61] Wiklund 2008 [63]		
							Belanger-Levesque 2014 [37]	**Experience of witnessing assisted vaginal delivery (partners) (Low confidence)** Witnessing an emergency CS or instrumental birth seems to be associated with less positive reports of childbirth for partners than a spontaneous vaginal birth. Emergency CS seems to be associated with marginally higher scores than instrumental birth, but only two studies measure this comparison. In one study, partners reported having panic attacks during the birth, and a few said they wouldn’t have more children. Some would prefer their partner chose an elective cs next time.	
							Chan 2002 [38]		
							Hildingsson 2013 [28]		

### What views, beliefs, concerns and experiences have been reported in relation to AVD?

Women’s experiences of assisted vaginal delivery (Table 2) were reported in 16 surveys [28, 29, 36, 43, 44, 46–48, 51, 53, 56, 58, 60–63]. Only one of these was from a LMIC country (Uganda) [29]. In these surveys, having an unplanned mode of birth, emergency CS or AVD (and especially where the intervention is done for delay in labour rather than for acute clinical risk) seemed to be associated with less positive reports of childbirth experience for women. A better experience was reported after ventouse than forceps in most, but not all comparisons. Instrumental birth with episiotomy was the most distressing, especially after trial of labour following previous CS. Further detail as to why and how the unplanned nature of AVD impacts on women’s experiences was evident in the theme *Coming to know AVD by experience* (Table 4). The emergent theme A birth you couldn’t plan for encapsulates postnatal mothers’ and fathers’ concerns (in HICs) relating to Expectations and preparedness for AVD [23, 26]. In part, this was because AVD had simply not been contemplated beforehand or did not fit into women’s ideas of what birth would be like: “*I sort of missed out the forceps and ventouse, in my mind I’d sort of thought it was going to be a natural delivery or caesarean, so I hadn’t really considered forceps or ventouse*” [23]. In addition to views of feeling unprepared, the belief that AVD could not be prepared for was also evident. Some participants felt disillusioned because of the disparity between their birth plans and what happened. In two UK studies there were views that AVD was not adequately explained in antenatal education. Other women, however, described deliberately avoiding consideration of the possibility, in order to manage their own feelings about birth: reading too much information was believed to provoke anxiety. Women and men in two UK studies described ‘keeping an open mind’: believing that, with regard to birth, “*There are so many variables that no one can predict”* [26]. In the same two qualitative studies [23, 26], both from the UK, mothers’ and fathers’ Beliefs about need/indications influenced their acceptance of the procedure: “*Surprisingly to me I was quite happy to go along with the doctor’s call. I normally would question why and how but at the time it seemed like an emergency*” [26]. However, findings from these two studies also suggested there could be lack of understanding about why an AVD had been performed. Some women expressed beliefs that there had been problems with their baby that necessitated AVD, while others described being unable to recall why they had had an AVD. Reconciling/coping with experience emerged as a theme in one study from the UK [26]. Finding a context for their birth experiences, believing it to be necessary for the baby or seeing the baby’s wellbeing as a ‘*priority’*, allowed women to come to terms with their birth experience, while other women were unable to reconcile.

Fourteen surveys contributed to the quantitative narrative review finding reporting the Impact of assisted vaginal delivery (women) (Table 2) [2, 28, 29, 36, 38, 40, 42–44, 54, 55, 58, 61, 63]. Studies have variously measured postnatal mood, sexual function, desire to have more children, dyspareunia, postnatal fear of childbirth, pain, haemorrhoids, backache. Unsurprisingly, having an emergency CS or an AVD appeared to be associated with less positive outcomes than having a spontaneous vaginal birth or an elective CS. Having a spontaneous vaginal birth without instruments or episiotomy seemed to result in the most positive outcomes in the short and longer term for most variables. In some studies, emergency CS was associated with least positive impacts, followed by assisted vaginal birth (negative outcomes reported for both forceps or ventouse in some studies – others show better outcomes for ventouse than CS in the short and longer term). In others, instrumental birth was the most distressing. Surveys that assessed preference for mode of birth next time indicate that spontaneous vaginal delivery is preferred by most, with some preferring planned CS. If instrumental birth is required, most seemed to prefer ventouse over forceps. For partners the experience of witnessing assisted vaginal delivery (Table 2), resulted in a few stating that they wouldn’t have more children, and some would prefer their partner chose elective CS next time [28, 37, 38]. There was agreement between this finding and the qualitative emergent theme Mixed views about any future pregnancy and delivery (Table 4) and the reasons for future preferences [23, 26, 31]. After the experience of AVD, some women were put off a future pregnancy, even if they perhaps would have wished for more children: *“I would like another baby but that is there at the back of my mind thinking oh could I really go through all that again”* [23]. Others wished to avoid the possibility of enduring AVD again by electing to have a caesarean section: “*I don’t want to have to go through all of that again ... I just wanna have one slice in the belly and whoosh!”* [23]. However, other women expressed the wish for vaginal birth if they were to become pregnant again, with some suggesting they would be more confident next time as they would feel prepared: *“If I have to have that with another baby it won’t ever be as worrying because I know exactly what to expect”* [23].

### What are the influencing factors (barriers) associated with low use of/acceptance of AVD?

Twelve surveys, three from LMICs [12, 41, 66] and nine from HICs [39, 45, 46, 50, 54, 55, 58, 61, 64] report prevalence rates by unit or by practitioners. At each time period, and where studies include a range of sites, Prevalence of assisted vaginal delivery (Table 2) varied widely. Lack of equipment and lack of trained staff were the prominent concerns perceived to contribute to low prevalence and early default to caesarean section. Reluctance to use AVD for some practitioners in one UK study was associated with insufficient pain relief for women, the presence of significant fetal caput, or lack of enough experience to become skilled [50]. In general, practitioners in more recent studies seem to be more positive about using the ventouse than about using forceps. One study investigating midwife ventouse practitioners in the UK noted that they were generally confident following their training in this technique, and that their extensive experience of spontaneous deliveries gave them confidence in, sometimes, not performing a ventouse when called, subsequent to estimating that the baby could safely be born spontaneously [34].

There were mixed findings about self-reported Skills (development) in assisted vaginal delivery of obstetricians in determining the need for, seeking a second opinion in, and accuracy of clinical skills for, instrumental delivery (Table 2) [39, 41, 43, 50, 57, 64]. Midwives who were trained in using ventouse in the UK seemed to be confident in its use [34]. Actual skills and competence were not tested in any included studies. The results of one relatively recent UK study [50] include professional views on use of ultrasound to assess fetal position prior to conducting AVD, showing 1:5 have used it, but including strong views that it should not become a replacement for clinical assessment skills. Professional attitudes to the use of assisted vaginal delivery varied by country, training programme, and seniority (Table 2) [39, 41, 45, 57, 59, 64]. In two UK surveys reporting the Personal attitudes to mode of birth for oneself/partner (obstetricians) the majority of respondents were happy to have an assisted vaginal birth, as an alternative to caesarean section for mid-cavity arrest, especially if they could choose the operator (Table 2) [35, 65]. As shown in Table 5 Convergence coding matrix, data relating to the use of AVD, health professionals’ skills, professional attitudes and personal attitudes, were not reported in any of the qualitative studies.

There was some evidence of the factors that influence women’s acceptance of the procedure in the qualitative theme turbulent feelings about the actual experience (Table 4), which describes the powerful and contrasting feelings women and men experience in relation to AVD. In five qualitative studies, from three countries (all HICs), women and men used strong imagery to convey their Frightening and violent experiences of AVD [26, 30–33]. Women were distressed when the procedure was carried out in a way they experienced as lacking care or compassion: *“The doctor came in and just basically ripped her out with forceps, it’s just like extracted her from my body. I really think part of it was the position ... all these people in there and the total lack of... that there was a human being on the table [ crying] going through this*” [33]. Men and women were also distressed by the perception of AVD as a violent experience for the baby: there were fears about injury to the baby, and feelings of shock at the forcefulness of the procedure: “*I honestly expected to see the baby’s head dangling from the end [-] sounds horrible but that’s the amount of force and then the noise of the pop and then seeing the doctor hit the wall and then the mess that followed it was something out of a horror film”* [26]. Some women reported experiences of detachment or dissociation, being physically present but mentally absent: “*Actually, I was totally gone, I know there are tons of people in the room and they asked me simple stuff but I couldn’t even answer*” [30]. In three surveys (all HICs) the Experience of witnessing assisted vaginal delivery (partners) seemed to be associated with less positive reports of childbirth for partners than spontaneous vaginal birth (Table 2) [28, 37, 38].

In the 16 surveys of Women’s experiences of assisted vaginal delivery, a few studies reported that negative experience of AVD is associated with poor pain relief (Table 2) [28, 29, 36, 43, 44, 46–48, 51, 53, 56, 58, 60–63]. However, in one study women with spontaneous birth compared to AVD reported more problems with postpartum pain, and intrapartum pain management [58]. Contradictory views about Pain was also an emergent theme from four qualitative studies (all from HIC; Table 4) [26, 30–32]. For some women, effective pain relief enabled them to feel ‘*relaxed’* about the prospect of AVD, while others described feeling supported to manage pain: “*They really listened to how I felt and how I wanted things when I was in pain and everything*” [30]. However, for some women, the pain was a traumatic experience “*When they were going to put in the vacuum extractor it was without doubt the worst thing I’ve ever been through; it was the worst thing I’ve ever done because it hurt so unbelievably much. So that I screamed right out, No way! Help, help, I’m dying.”[screams]* [32].

Also encompassed in the qualitative theme turbulent feelings about the actual experience were Positive or beneficial reactions to AVD expressed by women and men (Table 4) [26, 31, 32]. These views were evident in three studies, from two countries (both HIC), and conveyed feelings of relief that labour was over and that the baby had been born safely: “*Relief of an end to labour When it [the vacuum extractor] was attached, it was no problem and when she [the baby] came, everything was over and it just felt good”* [32]. Some women and men in one study reported simply feeling unperturbed: the process was as they had anticipated and they were not troubled by it. Some men in two studies from two countries described feelings of joy at the arrival of the new baby: *“I was really touched. That was one of the greatest moments in my life”* [31]. Also from these two studies, some men saw it as their role to provide emotional strength to support their partners, to stay ‘*calm’* so that their partners did not panic, or to help relay information from healthcare providers. While some felt unable to give as much support in the way they wished, others described coping with their own anxiety so that they could help.

### What are the enabling factors associated with increased appropriate use of/acceptance of AVD?

Six surveys (five HICs [36, 43, 50, 52, 61], 1 LMICs [41]) reported the importance of Communication, information and consent (Table 2) to women’s perceptions of their experience of AVD, with some evidence that many women do not have information about the risks and benefits of AVD (plus or minus episiotomy), either antenatally, intrapartum when the procedure is used, or postnatally to explain what happened. There was partial agreement between this quantitative finding and the qualitative theme Trust, control and relationships, which suggests that acceptance of AVD is facilitated by positive interactions with staff, respectful care, ongoing communication and trust in care providers when women’s control over birth is lost, while negative interactions with staff, poor communication, little involvement in decision-making and mistrust of caregiver is a barrier to acceptance (Table 4).

In three studies from two HICs, both women and men expressed a wish to be part of a team with healthcare providers describing how they welcomed Active participation through collaboration and involvement (Table 4) [26, 30, 31]. Healthcare providers could facilitate a collaborative approach both through their interactions “*She touched my belly and kind of helped me, now I think it feels like a contraction and now it’s time to push*” [30] and by involving women and men in decision-making. Men in one study expressed a wish to be included, and could feel excluded or that their experience was not recognised: *“OK you maybe not pushing the baby out but you are certainly going through the same if you take the physical aspect out going through the same emotions.”* [26]. Balancing control and trust between women, fathers and health professionals was reported to be important in five of six qualitative studies (Table 4) [26, 30–33]. In five studies from three countries women described feeling loss of control; this was experienced as challenging. Loss of control could be experienced as loss of physical control, or as lack of agency, with some women recalling feelings of hopelessness. A trusting relationship with healthcare providers enabled women to accept AVD and manage feeling out of control. *“People listening to what I said and acknowledging what it was like for me being kind made it easier for me to say right ok [-*] *completely trusted certainly the two midwives who were in the delivery room.”* [26]. Some men in one study described an erosion of trust as they began to feel communication from healthcare providers was not honest. “*We felt both of us after a while that it almost went to an extreme; when she started pushing and said like ‘wow’ almost after every contraction. They did not say that this would take a long time or a vacuum extraction would be needed, although they perhaps saw it... Finally you do not trust them so much*” [31].

The need to understand and to be understood was also an emergent theme that contributed to acceptance of AVD (Table 4) [26, 30, 31]. Participants in three studies from two countries talked about the importance of feeling heard and understood, and having their wishes taken into account: *“they listened so much and took things at my pace, so wait a little, I decided everything, they helped and gave me advice. It wasn’t as if they do this every day, it was as though I had to teach them. They really listened to how I felt and how I wanted things when I was in pain and everything”* [30]. Women valued acknowledgement of how they were feeling. Good communication was seen as reciprocal: in one study women emphasised the importance of explanations and information to facilitate involvement. Communication was described as an embodied process, with participants explaining how healthcare providers made eye contact with them or touched them.

As already stated, there were no qualitative studies to compare with quantitative findings reporting prevalence of AVD use in practice or skills and attitudes of staff (Table 5). However, the silences, agreement, and dissonances between quantitative data from different resource settings, are of note. In agreement with the studies from HICs, one study of obstetricians’ views in Egypt found significant differences (< 0.05) intheir acceptance of instrumental delivery based on professional level of seniority. Consultants’ attitudes were more favourable to AVD than specialists or registrars [59]. There was dissonance between studies from HICs and LMICs as to why AVD use may have declined. Some participants in HICs referred to changing obstetric fashions, whereas a study from Tanzania disputed the suggestion that vacuum extraction is not modern obstetrics, with the claim that the high incidence of HIV/AIDS could be the primary barrier [66]. In both HIC and LMIC settings, there was evidence of midwives performing AVD [12, 34]. Ugandan women in one study [29] reported similar views to women participants in HICs [26, 31, 32] in terms of turbulent feelings about the actual experience and mixed views about any future pregnancy and delivery. In addition, women in Uganda voiced concerns about the likelihood of their death and death of their baby associated with caesarean section, and with the financial cost of the operation. These concerns meant that assisted vaginal delivery was preferable.

## Discussion

Our mixed methods review identified only six qualitative studies of women’s views of AVD, and only one mixed-method study with qualitative data on provider views. We identified no studies of this design in low and middle-income countries. We included 36 studies in a quantitative narrative synthesis. Thirty-six percent of the studies did not differentiate between forceps and ventouse. In studies where the type of instrument was differentiated, there tended to be differences, usually (but not always) in favour of the ventouse. This suggests that future studies of mode of birth should always record which instrument was used, as not doing so limits understanding about what might work in particular circumstances, for particular women and practitioners. In quantitative surveys, in 33% of cases, women were asked about their experiences while still on the postnatal ward. In the study by Nolens et al. [29] in Uganda, women’s views about mode of birth did not change between 1 day and 6 months postnatally. However, other studies suggest that women tend to rate their experiences of labour and birth more positively as the postnatal period progresses [67] except for women who had extreme pain during labour and an epidural, many of whom continue to recall their birth negatively over time [68]. There is some evidence that this change in perception may be less positive for certain modes of birth, and notably CS with general analgesia [67]. These findings suggest that studies of women’s views of different modes of birth during the very early postnatal period may not be representative of their views and choices later. This may have particular resonance if women’s early views and experiences are seen as a proxy for preferred mode of birth for subsequent pregnancies.

Where outcomes were assessed by mode of birth in longitudinal surveys, spontaneous vaginal birth almost always resulted in lower levels of longer term physical and psychological harms, and more positive birth experience and self-esteem ratings from women. Planned caesarean section also tended to score relatively well on these measures. Women tended to report the most negative scores when they had had an emergency CS. On most measures assessed in the studies assessing various experience measures, women who had AVD were usually more positive than those who had an emergency CS, but less so than those who had either spontaneous vaginal birth or planned CS. This finding is unsurprising, as the reasons for using an instrument to assist birth or conduct an emergency CS would, by themselves, be a source of anxiety and affect women’s experiences. There is also a need to go beyond intrinsic aspects of AVD or CS, because the experience of (a trial of) ventouse, forceps and emergency CS are not mutually exclusive. In fact, the key and consistent insight emerging from the triangulation between qualitative and quantitative evidence women and their partners was the shock of the unexpected nature of events, the inherently unpredictable experience of birth by AVD (and, indeed, by emergency CS), and, particularly in high-income settings, the unmet expectations.

Respectful and relational factors that might mitigate this shock, and limit any consequent distress and adverse sequelae, also emerged strongly from both data sets. This review suggests that positive relationships, good communication, involvement in decision-making, and, for women and partners, (believing in) the reason for intervention were important mediators of birth experience, and thus may be of considerable value to alleviate emotional distress when complications arise that require an AVD or emergency CS. These findings resonate strongly with the growing literature on positive childbirth experiences [69] and on the value of respectful, kind, compassionate maternity care in general [70]. For both parents, it seems the distress of unexpected interventions associated with AVD (including episiotomy, need for unplanned pain relief, such as epidural analgesia, and concern for possible iatrogenic harm to the baby of using instruments) may be mitigated by how health professionals communicate, both at the time of decision-making, and during the process. Underlying expectations can also influence interpretation of the AVD experience. In our qualitative findings from HICs, it was apparent that women’s expectations and birth plans did not always anticipate the unpredictable nature of birth. This finding cannot be generalised to LMICs where women’s expectations of birth are different. In LMICs, in some contexts, AVD was preferred over CS due to fear of death of mother or baby subsequent to a surgical procedure, but this preference was less pronounced in HICs. Some survey data indicated highly negative experiences for partners, but most of the qualitative studies that included partners reported a more even mix of negative and positive accounts.

Prevalence data suggest that the use of AVD was much more common (and that experience with it was therefore much more mainstream) prior to 2000 than in the last decade or so. This was true for both high and low income settings. However there is variation between settings, with ventouse is still used regularly in some European countries. Professional attitudes and skills (existing skills, or the development of skills de novo) were simultaneously barriers and facilitators of AVD in quantitative studies. Our findings are consonant with other studies focussing on provider competencies. A 2015 study evaluated the impact of a 2-day training course called Advanced Life Support in Obstetrics (ALSO), designed to increase care providers’ capabilities in managing obstetric emergences, in four low-income countries [71]. After training, rates of vacuum deliveries increased in hospitals in the two countries where this was evaluated (Honduras and Tanzania). Two studies excluded after full text screening [72, 73] addressed issues of skills, both in high-income settings. The UK-based study by Bahl and colleagues used interviews and video recordings of expert midwives and obstetricians to understand non-technical skills involved in an AVD and identified seven main categories (situational awareness, decision making, task management, team work and communication, relationship with the woman, maintaining professional behaviour, and cross monitoring of performance) [72]. Simpson and colleagues in Canada used videos of expert clinicians performing simulated forceps deliveries to identify verbal and non-verbal components of performing a safe delivery [73]. Building skills by training and preparing providers in adequate decision-making for instrumental vaginal delivery is fundamental to increase the use safely and appropriately. However, the most effective modality, duration and frequency warrants further research [74–76]. After our analysis was complete, we identified two surveys, both from HICs (UK and Australia), of trainee obstetricians’ views on using Kielland’s forceps [77, 78], and a study by Bahl and colleagues from the UK [79] on decision making in instrumental delivery, which we would have included in our analysis had we identified them at the search stage. Bahl et al. used qualitative data to identify a sequence of decision points used by expert obstetricians in proceeding to an instrumental birth [79]. Both surveys of trainees found that low numbers of trainees had seen a forceps delivery [77, 78]. In the UK study, a majority of trainees said they would use forceps if trained, and expressed a wish to undertake training [77], while very few trainees in the Australian study expressed an intention to use forceps as a consultant [78]. These additional papers would not have altered our findings. However given our findings highlighting the importance of training, we are undertaking a systematic review of the limitations, barriers and potential facilitating factors relating to expertise, training and competencies in AVD.

The use of a systematic approach to evidence synthesis and the GRADE-CERqual tool for the summaries of findings from both qualitative and quantitative studies has ensured the robustness and applicability of our findings. Few qualitative studies were identified, and they were only from high income countries. This is an important limitation, as our qualitative findings alone cannot be assumed to reflect views and experiences of staff or parents in other settings, and the small number of studies and countries limits confidence in the review findings even within high income settings. However, a strength of this sequential mixed-methods review is that it combines evidence from both qualitative and quantitative studies. Previously, survey data has usually slipped through the inclusion net of both meta-analytic systematic reviews and qualitative evidence syntheses. The inclusion and systematic quality assessment and analysis of good quality surveys and audits in this review, and of the narrative findings emerging from them, is a methodological advance in this area. There are more data from quantitative surveys and audits, and more of these studies were based in LMIC settings. Thirteen studies reported on prevalence [12, 29, 39, 41, 45, 46, 50, 54, 64, 66], but two of them were undertaken before 2000, so they provide data for historical comparison rather than insights into current practice [45, 46]. Confidence in the findings statements was generally rated moderate (7/10 SoFs) for the qualitative papers, and moderate or low for the quantitative studies.

Going forward, it is important for researchers, guideline developers, policy makers to differentiate between ventouse, forceps, and spontaneous vaginal birth – these are often all referred to as “vaginal birth” despite being distinct clinically and experientially. It is also essential that we do not further dichotomise discussions about mode of birth (as either vaginal or caesarean), but conceive birth as a trajectory, educating women and families that AVD is an option during labour. How best to educate women without provoking anxiety remains an important research question. Attempts to increase the use of AVD to reduce unnecessary caesareans must be carefully grounded in an understanding of the local context, resources, practitioner skills and training, and the prior views and experiences of the local childbearing population. Training in the physiology, anatomy and mechanisms of straightforward birth, and the interaction of the mother/child dyad in labour, is critical to reduce poor decision making about the need for instrumental or surgical birth, and to improve understanding and techniques when AVD is required. Assessment of the impact of introducing AVD programmes into any setting (HIC or LMIC) should be undertaken with careful audit of the views, experiences, confidence and competence of staff at the outset, and again when they have built skills, experience and confidence. Training of midwives to undertake AVD warrants further research, as their skills and experience in managing uncomplicated vaginal births places them in an optimal position for appropriate decision-making and use of the instrument. Audit of views, experiences and outcomes of women, partners and birth companions should continue into the longer term, and not just be undertaken on the postnatal ward.

## Conclusions

Views and experiences of AVD are complex and varied. Although reports of traumatising experiences are numerous, experiences and views on AVD are driven to some extent by anxiety and distress due to the unexpected nature of the event. Information, positive interaction and communication with providers, and respectful care are facilitators for acceptance of AVD. Barriers include lack of training and skills for decision-making and use of instruments. Expanding AVD use must be preceded by high quality training and skills development in the recognition of both the physiology and the pathology of labour progress and maternal/fetal wellbeing, as well as in the assessment for, and use of, AVD techniques to ensure minimum trauma for mother and baby. Local resources to enable safe use and optimum short and longer-term outcomes of AVD and accompanying procedures (such as episiotomy) are essential, both for childbearing women, and, where they are present, for their birth companions.

## Supplementary information

**Additional file 1.** Search strategy Ovid medline.

## Data Availability

All data generated or analysed during this study are included in this published article [and its supplementary information files].

## Abbreviations

AVD: Assisted vaginal delivery
CS: Caesarean section
HIC: High income countries
LMIC: Low and middle income countries
WHO: World Health Organization
