# Supplementary material for: Women’s, partners’ and healthcare providers’ views and experiences of assisted vaginal birth: a systematic mixed methods review
Source: Reprod Health. 2020 Jun 1;17:83. doi: 10.1186/s12978-020-00915-w (PMC7268509; doi:10.1186/s12978-020-00915-w)
Supplement: Supplementary file 1 — Additional file 1. Search strategy Ovid medline. [file 12978_2020_915_MOESM1_ESM.docx]

Additional File 1

Search strategy Ovid medline

1. (woman or women or mother or mothers or father or fathers or birth companion or doula or midwife or midwives or nurse-midwife or nurse-midwives or traditional birth attendant or obstetrician or physician or obstetric nurse or policy makers or prenatal educator).ab.

2. (assisted vaginal delivery or ventouse or vacuum extraction or vacuum-assisted delivery or obstetrical extraction or forceps delivery or instrumental delivery or Kiwi).ab.

3. (views or experiences or opinions or acceptability or clinical decision making or attitudes or facilitators or enablers or barriers or Attitude of Health Personnel or satisfaction).ab.

4. 1 and 2 and 3
